# Supplementary figures and images for: Pan-cancer analysis and validation show GTF2E2’s diagnostic, prognostic, and immunological roles in regulating ferroptosis in endometrial cancer
Source: PLoS One. 2025 Apr 23;20(4):e0321983. doi: 10.1371/journal.pone.0321983 (PMC12017540; doi:10.1371/journal.pone.0321983)

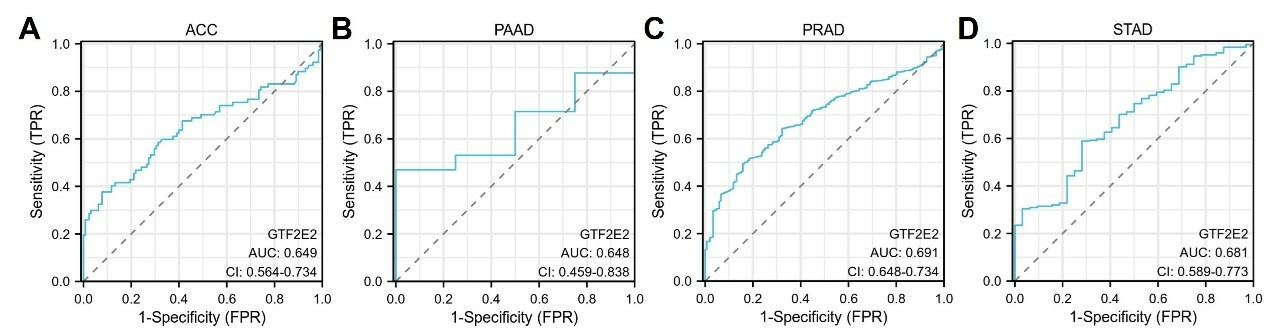

Supplement: S1 Fig — Cancers with AUC < 0.7 for GTF2E2: (A) ACC, (B) PAAD, (C) PRAD, (D) STAD. (TIF) [file pone.0321983.s001.tif]

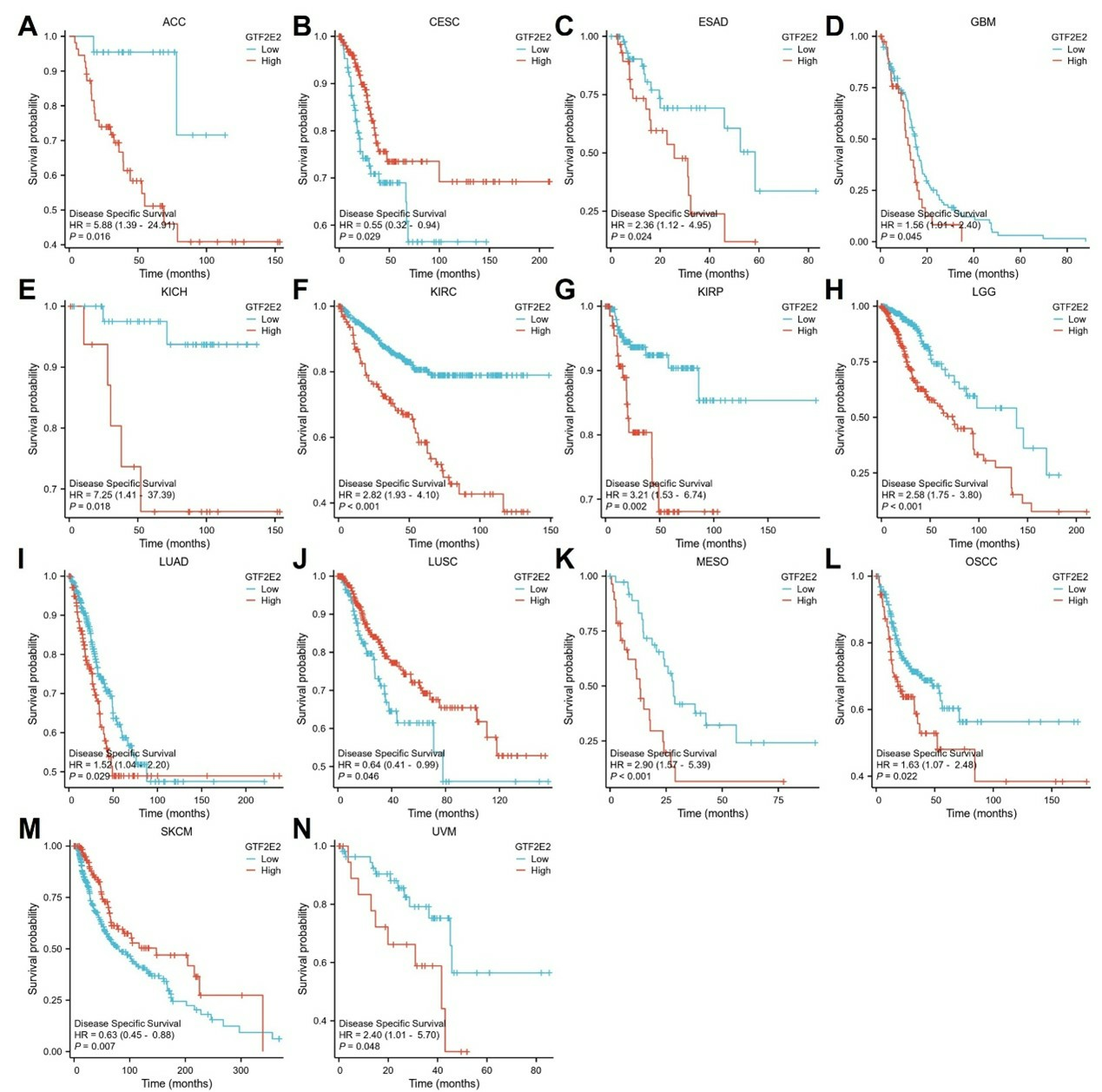

Supplement: S2 Fig — DSS K-M curve for GTF2E2 14 cancer types. The unit of X-axis is month. (A) ACC, (B) CESC, (C) ESAD, (D) GBM, (E) KICH, (F) KIRC, (G) KIRP, (H) LGG, (I) LUAD, (J) LUSC, (K) MESO, (L) OSCC, (M) SKCM, (N) UVM. (TIF) [file pone.0321983.s002.tif]

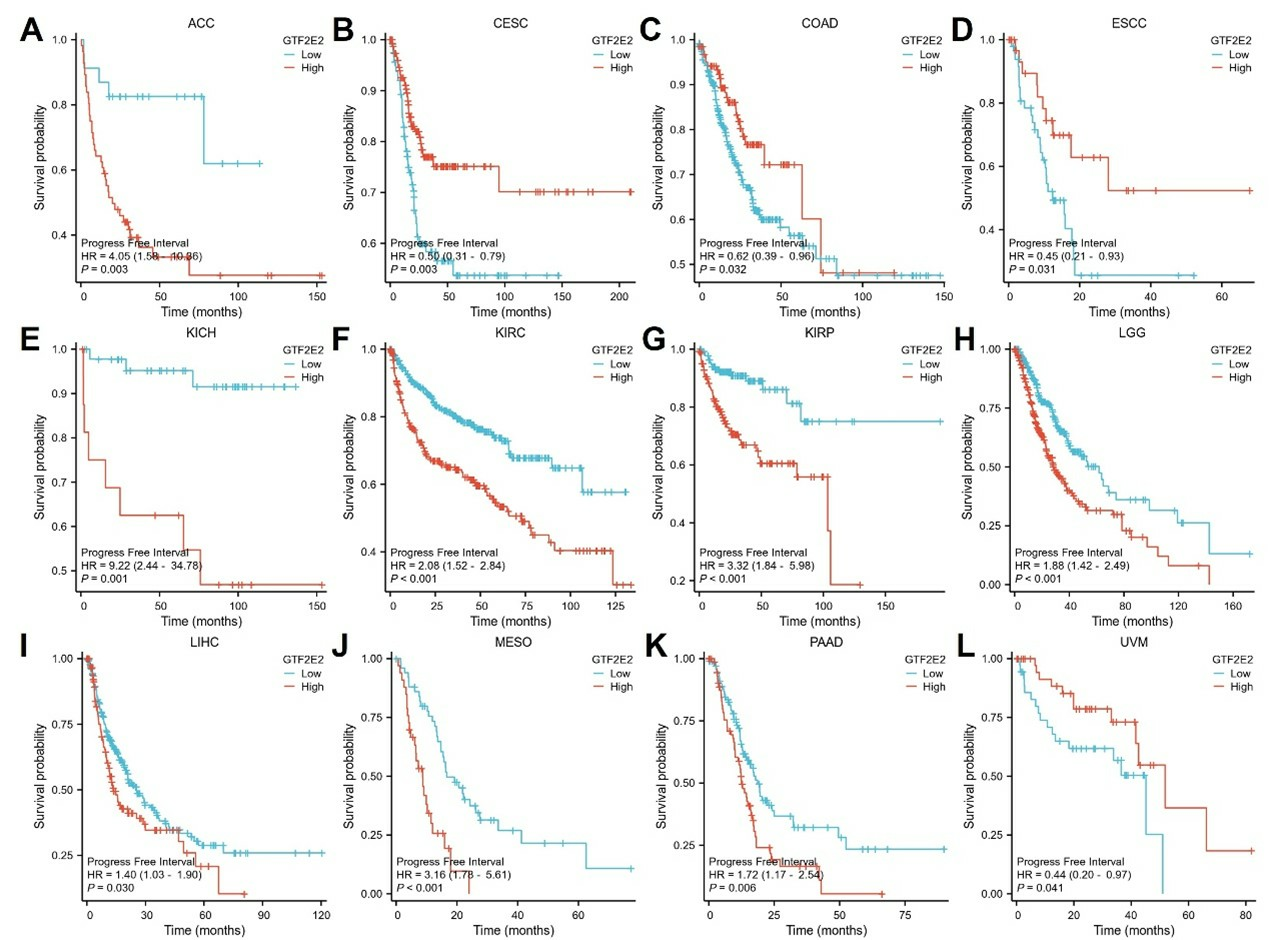

Supplement: S3 Fig — PFI K-M curve for GTF2E2 12 cancer types. The unit of X-axis is month. (A) ACC, (B) CESC, (C) COAD, (D) ESCC, (E) KICH, (F) KIRC, (G) KIRP, (H) LGG, (I) LIHC, (J) MESO, (K) PAAD, (L) UVM. (TIF) [file pone.0321983.s003.tif]

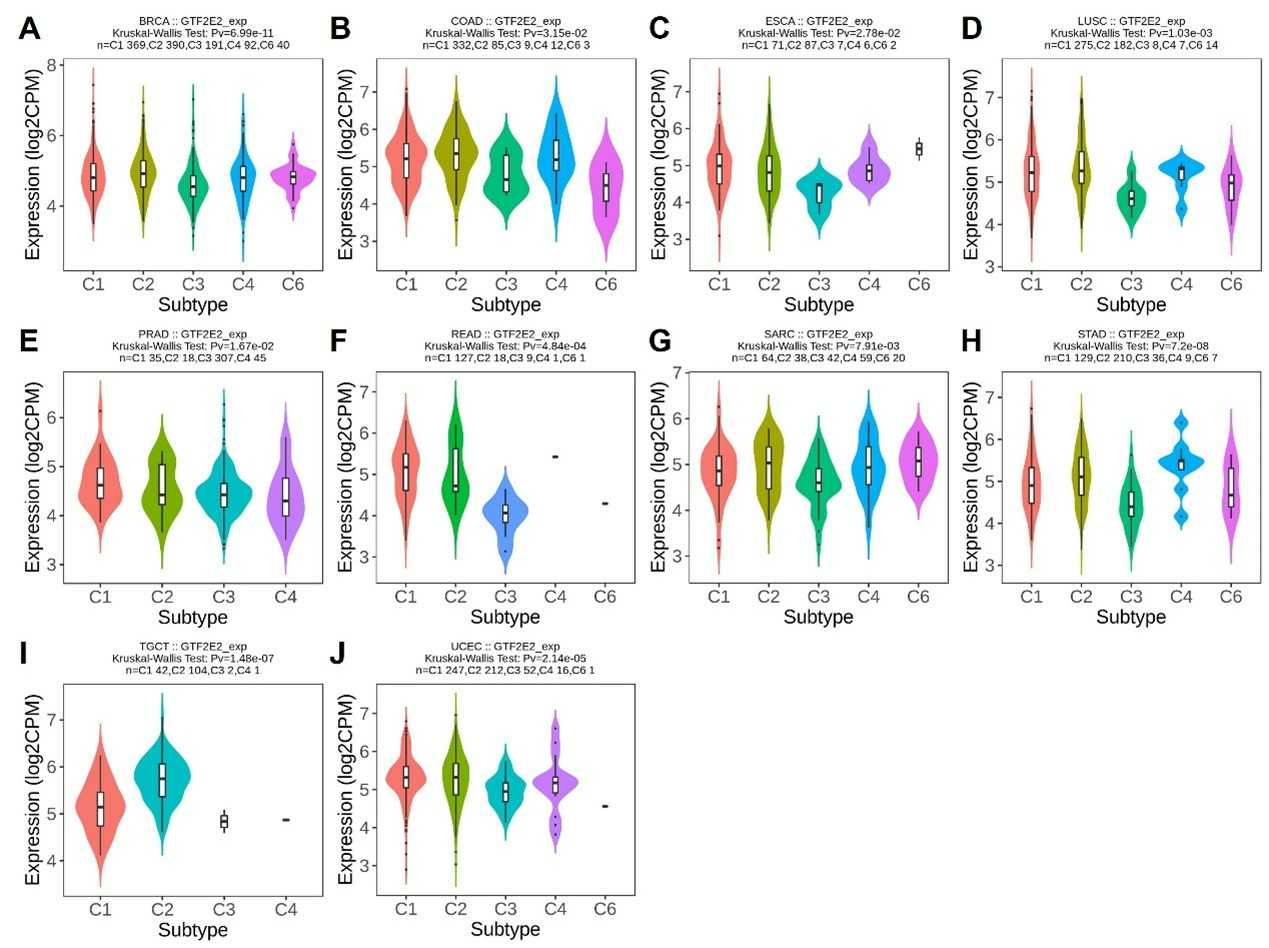

Supplement: S4 Fig — (A) BRCA, (B) COAD, (C) ESCA, (D) LUSC, (E) PRAD, (F) READ, (G) SARC, (H) STAD, (I) TGCT, (J) UCEC. (TIF) [file pone.0321983.s004.tif]
