# Supplementary material for: Pan-cancer analysis and validation show GTF2E2’s diagnostic, prognostic, and immunological roles in regulating ferroptosis in endometrial cancer
Source: PLoS One. 2025 Apr 23;20(4):e0321983. doi: 10.1371/journal.pone.0321983 (PMC12017540; doi:10.1371/journal.pone.0321983)
Supplement: Attachment — Submitted filename: Response_to_Reviewers_auresp_2.docx [file pone.0321983.s007.docx]

We sincerely thank the editor and all reviewers for their valuable feedback that we have used to improve the quality of our manuscript (Submission ID: PONE-D-24-58020). The reviewers’ comments are shown in italics below, with specific issues numbered. Our responses are provided in regular font.

***Comments from Reviewer:***

*1. The author stated in the revision comments that the images for Figure 3 (G, K, J) had been replaced, but the submitted revised figures show no differences from the original ones. Please verify the figures.*

**The author’s answer:**

Thank you very much for taking the time to review our manuscript and for providing invaluable feedback regarding the images in Figure 3 (G, K, J). We truly appreciate your thorough assessment and the opportunity to clarify our work.

We wish to explain that our immunohistochemistry (IHC) results for GTF2E2 were obtained from the publicly available Human Protein Atlas (HPA) database (https://www.proteinatlas.org/). Following your comments, we re-examined the HPA data for both normal and cancer tissues of KIRC, THCA, and STAD. Upon careful review, we found that the differences in GTF2E2 expression in these tissues were indeed not very pronounced. Realizing that retaining these images might cause confusion and potentially mislead readers regarding the clarity of GTF2E2 expression, we have decided to remove the IHC images for KIRC, THCA, and STAD.

We confirm that removing these images does not affect our overall findings or conclusions. Nonetheless, we sincerely apologize for any inconvenience or confusion this may have caused. We take full responsibility for our oversight and lack of rigor in verifying the images before resubmission. Your comments have prompted us to scrutinize our figures more carefully, and we have taken the necessary steps to ensure the accuracy and clarity of our data going forward.

Thank you again for your constructive suggestions and for giving us the opportunity to improve our manuscript. We appreciate your understanding and patience, and we remain committed to maintaining the highest standards of research and publication ethics.
